# Interrogation of a live-attenuated enterotoxigenic *Escherichia coli* vaccine highlights features unique to wild-type infection

**DOI:** 10.1038/s41541-019-0131-7

**Published:** 2019-08-28

**Authors:** Subhra Chakraborty, Arlo Randall, Tim J. Vickers, Doug Molina, Clayton D. Harro, Barbara DeNearing, Jessica Brubaker, David A. Sack, A. Louis Bourgeois, Philip L. Felgner, Xiaowu Liang, Sachin Mani, Heather Wenzel, R. Reid Townsend, Petra E. Gilmore, Michael J. Darsley, David A. Rasko, James M. Fleckenstein

**Affiliations:** 1 Johns Hopkins Bloomberg School of Public Health, Baltimore, MD USA; 2 Antigen Discovery, Inc. (ADI), Irvine, CA USA; 3 Department of Medicine, Division of Infectious Diseases, Washington University School of Medicine, Saint Louis, MO USA; 4 Vaccine R & D Center, University of California, Irvine, Irvine, CA USA; 5 Enteric Vaccine Initiative, PATH, Washington DC, USA; 6 Department of Medicine, Divsion of Endocrinology, Metabolism and Lipid Research, Washington University School of Medicine, St. Louis, USA; 7 MD Biologic Consulting Ltd, Cambridge, UK; 8 The Institute for Genome Sciences, University of Maryland School of Medicine, Baltimore, MD USA; 9 Medicine Service, John Cochran VA Medical Center, St. Louis, MO USA

**Keywords:** Live attenuated vaccines, Bacterial host response, Bacterial infection, Gastroenteritis

## Abstract

Enterotoxigenic *Escherichia coli* (ETEC) infections are a common cause of severe diarrheal illness in low- and middle-income countries. The live-attenuated ACE527 ETEC vaccine, adjuvanted with double mutant heat-labile toxin (dmLT), affords clear but partial protection against ETEC challenge in human volunteers. Comparatively, initial wild-type ETEC challenge completely protects against severe diarrhea on homologous re-challenge. To investigate determinants of protection, vaccine antigen content was compared to wild-type ETEC, and proteome microarrays were used to assess immune responses following vaccination and ETEC challenge. Although molecular interrogation of the vaccine confirmed expression of targeted canonical antigens, relative to wild-type ETEC, vaccine strains were deficient in production of flagellar antigens, immotile, and lacked production of the EtpA adhesin. Similarly, vaccination ± dmLT elicited responses to targeted canonical antigens, but relative to wild-type challenge, vaccine responses to some potentially protective non-canonical antigens including EtpA and the YghJ metalloprotease were diminished or absent. These studies highlight important differences in vaccine and wild-type ETEC antigen content and call attention to distinct immunologic signatures that could inform investigation of correlates of protection, and guide vaccine antigen selection for these pathogens of global importance.

## Enterotoxigenic E. coli: deep dive into vaccine characteristics

Adjuvanted ACE527 is an experimental oral live-attenuated vaccine for enterotoxigenic *E. coli* (ETEC) but has been shown to elicit only partial protection. James M. Fleckenstein and colleagues perform a genomic and proteomic characterization of ACE527 and compare it to H10407 – a wildtype virulent strain of *E. coli* that can generate fully protective acquired immunity. While sharing many canonical antigens with wild-type ETEC, the attenuated *E. coli* strains composing ACE527 lack a number of critical characteristics, in particular they lack core components of the flagellar machinery or the adhesin EtpA and are immotile. Accordingly, and in contrast to wildtype challenge, vaccination with ACE527 fails to elicit robust antibody responses to these non-canonical antigens and this might underpin the incomplete protection afforded by this vaccine.

## Introduction

Enterotoxigenic *Escherichia coli* (ETEC) cause substantial morbidity due to diarrheal illness in resource-poor areas of the world where young children are disproportionately affected. In children under five years of age, these pathogens are among the leading causes of moderate-to-severe diarrhea and deaths due to acute diarrheal illness.^1,2^ ETEC also causes severe illness, clinically indistinguishable from cholera,^3–5^ and death in older individuals^6^ and remains the most common cause of travelers’ diarrhea. While oral rehydration therapy and other measures have contributed to a decline in deaths due to diarrheal illness, ETEC have been linked to post-diarrheal sequelae including malnutrition, growth stunting, and impaired cognitive development greatly compounding the impact of these infections.^7^

The ETEC pathovar is defined by the production and effective delivery of heat-stable (ST) and/or heat-labile (LT) enterotoxins to epithelial receptors in the small intestine. In the classical ETEC pathogenesis paradigm, plasmid-encoded colonization factor (CF) or coli surface (CS) antigens facilitate small intestinal colonization.^8^ Interaction with small intestinal enterocytes leads to toxin-induced alterations in salt and water transport that result in net fluid losses into the intestinal lumen and ensuing watery diarrheal illness ranging from mild to severe and cholera-like.^3^

ETEC infections among young children in endemic regions are thought to result in acquired immunity and a decreasing incidence of infection with age.^9^ Indeed, controlled human infection studies demonstrate that homologous re-challenge with the ETEC H10407 strain, which encodes CFA/I, results in robust protection against symptomatic ETEC infection.^10^ However, precise correlates of protection^11^ have not been established, and the majority of immunologic studies have focused on canonical virulence factors, namely the CF/CS antigens and heat-labile toxin. Nevertheless, recent studies indicate that the repertoire of immune responses following infection extends beyond these classical antigens.^12^

Because of inherent genetic plasticity of *E. coli*, no canonical virulence factor is universally conserved in ETEC. Therefore, to achieve broad coverage, most ETEC vaccines under development adopt a polyvalent approach targeting multiple CF/CS antigens and LT. ACE527, was developed as a live-attenuated vaccine combining three strains that collectively express CS1, CS2, CS3, CS5, CS6, CFA/I, and the B subunit of LT.^13^

In recent studies vaccination with ACE527 alone failed to protect against severe diarrhea upon challenge with H10407, while ACE527 adjuvanted with double mutant heat-labile toxin (dmLT)^14^ afforded significant protection (PE ~ 66%) (Clinical Trials Identifier NCT01739231).^15^ Comparatively, H10407 challenge elicits nearly complete protection against homologous re-challenge.^10^ To comprehensively assess the adaptive immune response to vaccination we examined the antigen continent of ACE527 and used ETEC protein microarrays to examine antibody responses to the vaccine ± dmLT. These responses were then compared to vaccine placebo controls and to challenge with H10407 to profile potential benchmarks of protection.

## Results

### Genomic and proteomic characterization of ACE527 vaccine strains

**Fig. 1:**
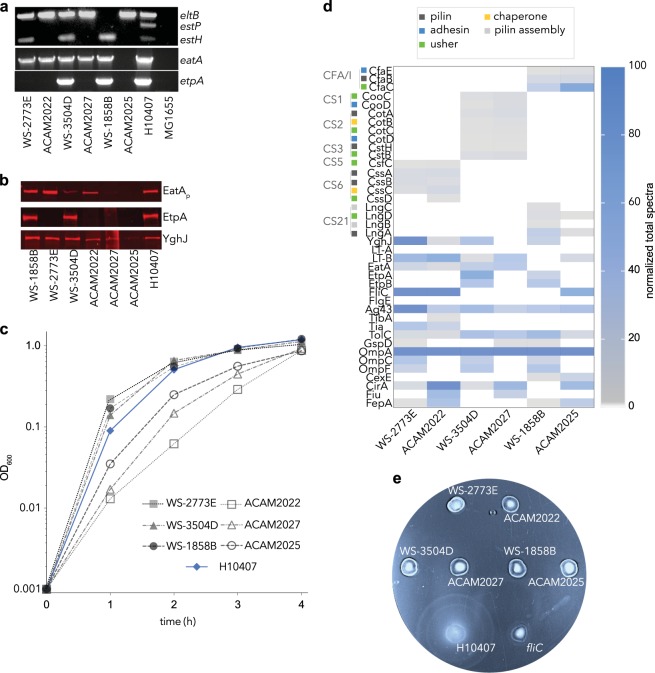
vaccine strain molecular characterization. **a** PCR confirmation of ACE527 genotypes. Shown are (top) multiplex enterotoxin (*eltB*, *estP*, and *estH*) PCR; (middle) *eatA* autotransporter gene; (bottom) *etpA* amplification from wild-type parental ETEC strains, corresponding live-attenuated ACE527 vaccine constructs, and controls. H10407 and MG1655 are shown at right as a positive and negative controls, respectively. **b** Immunoblots (TCA-precipitated culture supernatants) for secreted antigens EatA passenger domain (EatA_p_), the EtpA adhesin, and the YghJ metalloprotease. (Gel and blot images in (**a**) and (**b**) respectively were each derived from a single set of experiments). **c** Growth curves of parental strains (closed symbols) and corresponding vaccine strains (open symbols). H10407 growth curve (blue symbols) is shown for comparison. Summary of proteomic data from interrogation of ACE527 parents and vaccine strains. Parent strain and corresponding mutant are paired on the *X* axis. **d** Heat map values reflect the maximum normalized total spectra obtained from analysis of secretome, outer membrane and outer membrane vesicles. Colonization factors are grouped at the top of the map and color-coded on the *Y* axis by function. **e** ACE527 parental and vaccine strains are immotile in soft agar assays. H10407 (bottom left) and an isogenic *fliC* mutant (bottom right) are shown as positive and negative motility controls, respectively

Whole genome DNA sequence data was used to verify the genotypes of wild-type parental isolates and the engineering of the ACE527 strains (summarized in supplementary table 1). As anticipated,^13^ the three engineered vaccine strains, ACAM2022, ACAM2025, and ACAM2027, collectively encoded the full complement of six CF/CS antigens (CFA/I, CS1,CS2, CS3, CS5, and CS6), and each encoded both the B subunit of heat-labile toxin, as well as the type II secretion system (T2SS) responsible for export of both LT^16^ and YghJ^17^ (supplementary table 3). Two of the three parental strains, WS1858B and WS3504D were noted to contain the plasmid *etpBAC* locus which encodes the two-partner secretion system responsible for production and export of the EtpA adhesin,^18^ however analysis of the corresponding attenuated vaccine derivatives, ACAM2025 and ACAM2027 revealed that this locus had been lost in the vaccine strain construction (Fig. 1a, b). Similarly, the *eatA* gene which encodes a serine protease autotransporter protein that degrades MUC2 mucin,^19^ was present in each of the parents but absent from ACAM2025 (Fig. 1a, b).

The growth of each parental isolate and corresponding ACAM live-attenuated bacteria was compared to H10407. In general, the growth of the parental wild-type strains paralleled that of H10407, while the growth of ACAM vaccine strains lagged slightly, potentially reflecting the combined effect of OmpC and OmpF mutations on growth^20^ (Fig. 1c).

In keeping with the analysis of the individual genomes, mass spectrometry studies verified production of one or more features of the anticipated CF antigens, including outer membrane usher proteins, and major and minor pilin subunits, as well as LT-B in each of the vaccine strains. As anticipated, no EatA protein was detected in the supernatant of ACAM2025 and no EtpA was produced by the vaccine strains (Fig. 1d, supplementary dataset 2). Interestingly, we noted substantially increased expression of several iron-siderophore transport proteins including FepA, CirA, and Fiu in vaccine strains relative to the corresponding parents, possibly due to the absence of OmpC, and OmpF porins, and the complex regulation of the outer membrane architecture by small non-coding rRNAs known to govern the production of CirA and FepA.^21^

In contrast to recent studies of the H10407 proteome^12^ where the major protein subunit of flagella, flagellin (FliC, serotype H11), and the flagellar hook protein, FlgE were present in abundance, FliC was not universally detected in the ACE527 proteomes, and we were unable to detect the flagellar hook protein, FlgE (Fig. 1d). Despite the identification of the flagellar operons in the genomes of all of the parent and mutant strains, we found that none of the ACE527 or progenitor strains were motile (Fig. 1e).

Collectively, these molecular characterization studies demonstrated that the ACE527 vaccine strains express all of the canonical ETEC antigens targeted in construction of the vaccine. However, they lack some virulence properties previously identified in the H10407 challenge strain including motility, production of key flagellar antigens, and other immunogenic proteins including the secreted EtpA blood group A lectin-adhesin.^12,22^

### Differential immune responses to ETEC antigens after vaccination and challenge

Recent controlled human infection studies with the H10407 strain of ETEC demonstrated that previously naive volunteers mount strong mucosal antibody responses to classical vaccine targets including CFA/I and heat-labile toxin as well as a select number of molecules that have not been traditionally been targeted in vaccines.^12^ Here we set out to examine immune response to the classical antigens targeted in ACE527, and to a broadly inclusive ETEC proteome represented on microarrays incorporating more than 4000 proteins.

Mucosal antibody, particularly mucosal IgA, is felt to be an essential determinant of protection against ETEC, that follows natural infection. Antibody secreted by circulating gut-homing lymphocytes can be obtained from peripheral blood mononuclear cells (PBMC) following recent mucosal exposure to antigens from enteric pathogens.^23,24^ These antibody lymphocyte supernatants (ALS) serve as a convenient hallmark of recent mucosal antigen exposure. Therefore, we tested ALS samples from individuals who received ACE527 ± dmLT and placebo controls prior to vaccination, after vaccination, and following challenge with ETEC H10407.

### Response to classical vaccine antigens

**Fig. 2:**
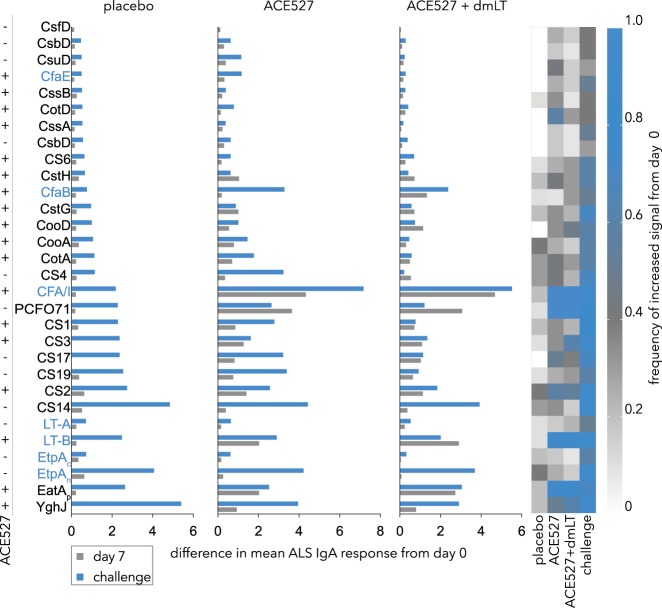
Immune response to purified colonization factor antigens and secreted ETEC virulence factors following vaccination and challenge. Bar graphs on left depict the difference in mean ALS IgA responses from day-1 to day 7 following vaccination with placebo, ACE527, and ACE527+ dmLT (gray bars), or following challenge with H10407 (blue bars). Antigens listed on the Y axis include all major colonization factors and subunits (top) and secreted virulence proteins LT (A and B subunits), EtpA (carboxy terminal repeat region EtpA_c_, and amino terminal secretion domain Etp_n_), EatA (passenger domain EatA_p_), and YghJ (bottom). The column on the far left indicates antigens present in (+) or absent from (−) the ACE527 vaccine strains. Antigens present in the H10407 challenge strain are depicted in Blue. Heat map at right depicts the proportion of volunteers exhibiting at least a 50% increase in signal intensity at day 7 following vaccination relative to baseline (day 0) values. Challenge values depicted are from day 7 following challenge of the placebo group with H10407

Following vaccination with either ACE527 or ACE527 adjuvanted with dmLT, we observed increases in ALS IgA immunoreactivity to most canonical antigens targeted in the vaccine. Seven days after vaccination, we found that each subject mounted robust responses to CFA/I, and the majority (≥75%) responded to B subunit of LT (Fig. 2, Table 1). In addition, we found strong responses in both groups to PCF071, with subjects responding this class 5b fimbrial antigen most closely related to CS1^25^ Table 1, supplementary fig. 1, while we observed more modest responses to CS17 another class 5b antigen, or CooD, a minor pilin subunit of CS1.^26^ More than half of vaccine recipients also mounted ALS IgA responses to CS2 and/or the CotA CS2 pilin in each of the vaccine groups. Responses to CS3 were strongest in the dmLT adjuvanted cohort where nearly 2/3 of recipients demonstrated increased responses following vaccination (Table 1). Altogether, these data demonstrate that vaccination with ACE527 with or without dmLT results in antibody responses to most classical antigens targeted in the vaccine. CS6 was an exception in this regard. Interestingly, an earlier analysis of samples from individuals immunized with ACE527, prepared from frozen lots of the vaccine, demonstrated a 13-fold increase in peak geometric mean ALS titers from baseline.^27^ Whether drying of the lyophilized preparation of the vaccine used in the present studies impacted the stability or presentation of the CS6 antigens to the recipients in the present study is presently unclear.

**Table 1:** Most differentially reactive purified protein antigens ALS IgA, responses day 0 to day 7 following ACE527 or ACE527 + dmLT

ACE527				ACE527 + dmLT			
Antigen	∆^a^	Frequency^b^	*p*	antigen	∆^a^	Frequency^b^	*p*
CFA/I	4.34	1.00	5.4 × 10^–6^	CFA/I	4.81	1.00	8.4 × 10^–7^
PCF071	3.66	1.00	4.1 × 10^–6^	PCF071	3.08	1.00	5.2 × 10^–7^
LT-B	2.04	0.75	3.2 × 10^–3^	EatAp	2.74	0.85	1.6 × 10^–4^
EatAp	1.62	0.75	7.8 × 10^–3^	LT-B	2.90	0.77	7.7 × 10^–4^
CS2	1.41	0.58	1.5 × 10^–2^	CS3^c^	1.09	0.62	2.0 × 10^–2^
YghJ	0.93	0.50	1.2 × 10^–2^	YghJ	0.80	0.62	3.0 × 10^–2^
CS17	0.82	0.50	3.4 × 10^–2^	CS2	1.14	0.54	1.7 × 10^–2^
CotA (CS2 pilin)	0.70	0.42	2.8 × 10^–2^	CooD	1.15	0.46	1.3 × 10^–2^
CsuD (CS14 adhesin)	0.39	0.42	2.1 × 10^–2^	CS17	1.04	0.38	3.9 × 10^–2^
CstH (CS3 pilin)	1.04	0.33	7.0 × 10^–2^	CfaB	1.34	0.31	3.0 × 10^–2^

^a^∆mean refers to difference in mean values at day 0 and day 7 with respect to the first vaccination

^b^Frequency refers to the proportion of subjects with at least a 50% increase in normalized signal intensity between day 0 and day 7 after the first vaccination

^c^Purified fimbriae

### Response to non-canonical antigens following vaccination and challenge

**Fig. 3:**
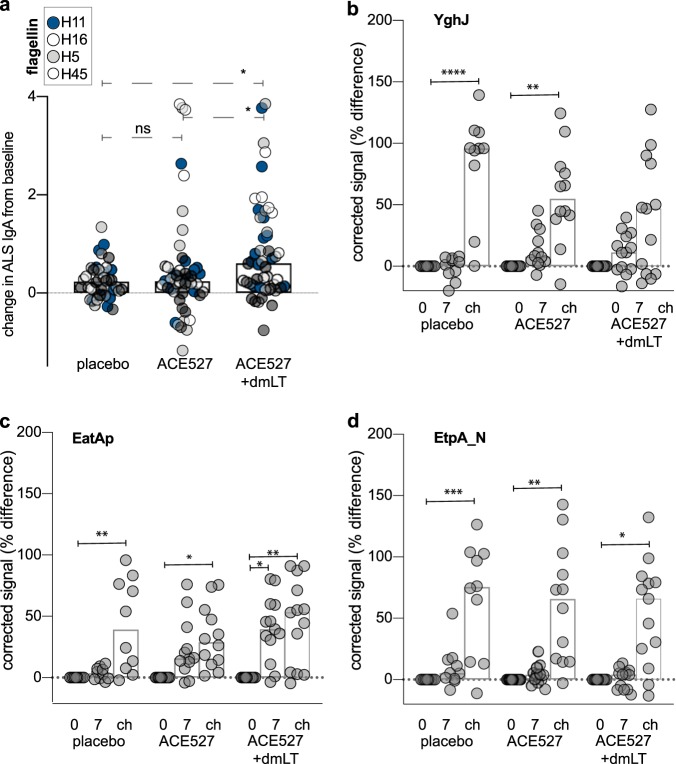
ALS IgA responses to non-canonical secreted antigens. **a** Comparison of ALS IgA responses against IVTT-produced flagellins representing the serotypes of the ACE527 strains (H5, H16, H45) and the H10407 challenge strain (H11). Shown are responses to three recombinant protein antigens: **b** YghJ, **c** the passenger domain of EatA (EatAp), and **d** the amino terminal region of EtpA (EtpA_N) secreted by the ETEC H10407 challenge strain and/or the ACE527 vaccine strains. Columns in each group represent data obtained at day 0, day 7 following vaccination, or day 7 following challenge with abbreviated labels of “0”, “7”, and “ch” respectively. Data in each vaccination or control group are represented as corrected for the baseline values obtained at day 0. Each symbol represents the change in normalized ALS IgA signal at baseline (day 0) to day 7 post immunization or after placebo administration. Values on the y-axis for B-D are expressed as the % difference of (signal-background)/background. (*<0.05, **<0.01, ***<0.001, **** ≤ 0.0001 by ANOVA comparison of normalized signal data with corresponding day 0 data without background correction; Kruskal–Wallis test for multiple comparisons). Open bars represent mean values

Although ACE527 was directed at classical ETEC CF/CS and LT-B vaccine antigens, recent studies indicate that following ETEC infection, subjects respond to antigens not targeted in canonical vaccine strategies. Following earlier H10407 challenge studies, we found that all subjects mounted robust responses to flagellin (FliC), the major protein subunit of flagella .^12^ Interestingly, however, consistent with poor production of flagellin and the lack of motility by the ACE527 vaccine strains, we found that fewer than 1/3 of the recipients of unadjuvanted ACE527 developed significant responses to FliC regardless of serotype. Notably, we found that following administration of the vaccine + dmLT the majority (92%) of recipients mounted responses to at least one flagellin molecule. Interestingly, these enhanced responses were not constrained to serotypes included in the vaccine as 10/13 subjects in the dmLT cohort responded to FliC from serotype H11 (Fig. 3a, Table 2) the serotype for H10407 challenge strain. As this serotype was not included in the vaccine itself, and responses to H-serotype specific regions of the flagellins represented in the vaccine were noticeably absent (supplementary fig. 2), the responses to FliC H11 and other flagellins may reflect recognition of the non-serotype specific highly conserved alpha helical regions comprising the amino and carboxy terminal regions of flagellin molecules (supplementary fig. 3).

**Table 2:** Most differentially reactive IVTT antigens ALS IgA, responses day 0 to day 7 following ACE527 or ACE527 + dmLT

ACE527				ACE527 + dmLT			
Antigen	∆^a^	Frequency^b^	*p*	Antigen	∆^a^	Frequency^b^	*p*
EatA _AA 533–1045_	2.62	0.75	1.6 × 10^–3^	EatA _AA 533–1045_	3.95	0.85	5.1 × 10^–5^
EatA _AA 1–1364_	0.98	0.33	3.8 × 10^–2^	EatA _AA 1–1364_	1.83	0.70	6.1 × 10^–4^
WS1858B FliC (H45)	0.90	0.25	6.8 × 10^–2^	Antigen 43	1.33	0.39	3.5 × 10^–2^
WS3504D_05115^c^	0.65	0.17	1.8 × 10^–1^	WS2773E FliC (H5)	1.18	0.69	2.8 × 10^–3^
CS3 pilin subunit A	0.65	0.17	1.7 × 10^–1^	H10407 FliC (H11)	1.16	0.80	1.9 ×10^–3^
Centroid_475810^c^	0.56	0.25	9.8 × 10^–2^	CfaB CFA/I pilin	1.04	0.30	6.5 × 10^–2^
WS2773E FliC (H5)	0.52	0.33	2 × 10^–1^	CS3 pilin	0.94	0.23	6.25 × 10^–2^
H10407 FliC (H11)	0.45	0.17	6.4 × 10^–2^	WS1858B FliC (H45)	0.90	0.46	6.6 × 10^–3^
Centroid_845698^d^	0.38	0.17	1.6 × 10^–1^	LT-A	0.81	0.39	1.7 × 10^–2^
Prophage integrase c577664	0.36	0.25	7.8 × 10^–2^	Transcriptional activator CadC	0.76	0.54	5.8 × 10^–3^
Transcriptional activator CadC	0.33	0.17	1.4 × 10^–1^	Aldehyde dehydrogenase PuuC	0.70	0.23	9.1 × 10^–2^

^a^∆mean refers to difference in mean values at day 0 prior to challenge and day 7 post challenge

^b^Frequency refers to the proportion of subjects with at least a 50% increase in normalized signal intensity between day 0 and day 7 after the first vaccination challenge

^c^Hypothetical protein

^d^Putative quorum sensing protein

We likewise observed differential responses to two secreted non-canonical antigens, the passenger domain of the EatA autotransporter protein, and YghJ a metalloprotease secreted by the type 2 secretion system. Both proteins were recognized following vaccination with ACE527 with 9/12 subjects responding to the EatAp and 6/12 responding to YghJ. These responses were enhanced slightly in the dmLT cohort (Table 1).

### Immune responses to non-canonical proteins predominate after challenge

In naive subjects and in vaccine recipients challenged with H10407, we noted enhanced responses to a number of proteins that were not recognized following vaccination. Among the most striking differential responses following challenge compared to vaccination were those to flagellar proteins. 9/10 placebo vaccinated subjects responded to both full length and the serotype specific region (AA174-399) of FliC H11 (Table 3). Likewise, 6/10 volunteers responded to the FlgE flagellar hook protein, a response that was absent in vaccinees prior to challenge, consistent with the lack of motility in the ACE527 strains and the absence of FlgE in the ACE527 proteomes.

**Table 3:** Differentially reactive IVTT antigens following ETEC H10407 challenge of naive subjects

Antigen	∆ mean^a^	Frequency^b^	*p*
FliC H11	6.06	0.90	5.8 × 10^–5^
Antigen 43	3.77	0.70	2.4 × 10^–3^
FliC H11 _AA 174–399_ (H11 serotype specific)	2.88	0.90	1.4 × 10^–4^
EatA passenger domain _AA 533–1045_	2.68	0.70	1.1 × 10^–2^
YghJ metalloprotease _AA 695–1493_	2.50	0.80	1.8 × 10^–3^
FlgE flagellar hook	1.51	0.60	2.8 × 10^–2^
OmpW	1.45	0.70	5.4 × 10^–3^
YghJ metalloprotease _AA 1–800_	1.19	0.70	4.3 × 10^–3^
AIDA-I family autotransproter YfaL	1.09	0.60	1.4 × 10^–2^
EatA serine protease _AA 1–1364_	0.99	0.40	3.9 × 10^–2^

^a^∆mean refers to difference in mean values at day 0 prior to challenge and day 7 post challenge

^b^Frequency refers to the proportion of subjects with at least a 50% increase in normalized signal intensity between day 0 and day 7 post challenge

In addition to flagellin, three secreted antigens YghJ (SslE),^28^ EatA,^19^ and EtpA,^29^ not currently targeted in classical ETEC vaccine approaches have been shown to be immunogenic in humans^12^ and associated with protection in animal models.^19,29–31^ Although we observed responses to YghJ following vaccination with either ACE527 or ACE527 + dmLT, the response to this protein was significantly increased following challenge with H10407 (Fig. 3b). Similarly, we observed the most robust responses to the EatA passenger domain following challenge with H10407 (Fig. 3c). In contrast, in keeping with the absence of EtpA in the three vaccine strains, vaccinees did not recognize EtpA following immunization, while both the placebo group and the vaccinated subjects mounted robust responses to EtpA upon challenge with H10407 (Fig. 3d, Table 4).

**Table 4:** Differentially reactive purified protein antigens following ETEC H10407 challenge of naive subjects

Antigen	∆ mean^a^	Frequency^b^	*p*
YghJ	5.40	0.90	1.1 × 10^–4^
CS14	4.85	0.90	1.8 × 10^–5^
EtpA amino-terminal domain	4.08	0.90	1.6 × 10^–3^
CS2	2.74	0.90	4.7 × 10^–3^
CS3	2.37	0.90	2.5 × 10^–3^
CS1	2.29	0.80	5.6 × 10^–3^
PCF071	2.29	0.80	1.9 × 10^–3^
EatAp	2.64	0.70	5.7 × 10^–3^
CS17	2.37	0.70	1.2 × 10^–2^
EtpA full length	1.34	0.70	8.1 × 10^–3^

^a^∆mean refers to difference in mean values at day 0 prior to challenge and day 7 post challenge

^b^Frequency refers to the proportion of subjects with at least a 50% increase in normalized signal intensity between day 0 and day 7 post challenge

## Discussion

A systematic appraisal of immune response to candidate vaccines can aid in vaccine optimization and identification of mechanistic correlates of protection. Here, we combined genomic and proteomic interrogation of a live-attenuated vaccine with immunoproteomic analysis following vaccination and experimental human challenge with ETEC H10407, an extensively characterized strain originally obtained from a patient with severe cholera-like diarrheal illness.^32^ Previous studies demonstrating that challenge with wild-type ETEC H10407 bacteria affords virtually complete protection against severe diarrhea on subsequent re-challenge^10^ provide important benchmarks for comparison of candidate vaccines. Importantly, the studies reported here highlight a number of features that distinguish the vaccine strains from the challenge strain and the corresponding immune responses.

First, although the wild-type isolates used to construct the vaccine had previously been serotyped for flagellar (H) antigens, we found that production of flagellin, the major subunit of flagella, by both the parent and the vaccine strains was deficient, and none were motile upon testing. Similarly, contrasting with our earlier analysis of the H10407 challenge strain,^12^ we were unable to detect FlgE, the flagellar hook protein, in either the parental or vaccine strains, suggesting that despite the presence of the genes required for assembly, early steps involved in biosynthesis of flagella are deficient^33,34^ in the isolates selected for engineering of ACE527. These deficiencies may have negatively impacted the protection afforded by the vaccine in a number of ways including loss of TLR5 mediated stimulation of innate immunity, and the potent adjuvant activity of flagellin. While H serotypes of ETEC vary considerably,^35^ motility is a highly conserved virulence characteristic, and the lack of motility of the vaccine strains likely resulted in suboptimal antigen delivery to sampling sites within the intestinal mucosa.^36^ In addition, highly conserved regions of flagellin that flank serotype specific regions of the molecule may not only serve as potent stimuli of innate immunity, but contain cross-protective epitopes.^29^ Intriguingly, addition of dmLT as a mucosal adjuvant significantly enhanced the response to multiple flagellins independent of whether the specific serotype was present in the vaccine.

The genomes of the three vaccine strains and parents also revealed that the *etpBAC* locus, which encodes the two-partner secretion system responsible for production and export of the EtpA adhesin,^18^ was present in two of the three parental strains but missing from the vaccine altogether, likely the result of engineering the vaccine strains to remove the plasmid-encoded toxins. EtpA is a high molecular weight glycoprotein secreted by ETEC that appears to facilitate bacterial adhesion by serving as a molecular bridge between flagella ^37^and the enterocyte surface where it binds to N-acetylgalactosamine (GalNAc) residues^38^ particularly when they are presented as the terminal glycan on human A blood group antigen. Interestingly, human challenge studies with H10407 demonstrate that individuals with A blood group are significantly more likely to experience severe diarrhea when challenged with this EtpA-producing strain.^22^ These and other recent studies^12^ demonstrate that EtpA is highly immunogenic and recognized by the majority of volunteers upon challenge with wild-type H10407, in distinct contrast to those immunized with ACE527 ± dmLT. Although EtpA is required for optimal delivery of both LT and ST enterotoxins, and is protective against ETEC infection in a murine model, further studies are needed to assess its role as a potential protective antigen in humans.

Protective immunity to ETEC is likely complex and may represent the cumulative response to *E. coli* core proteins, classical vaccine antigens, and more recently discovered proteins. Although the present studies, based on small numbers of human volunteers, do not permit us to establish clear mechanistic correlates of protection, they highlight the utility of combined genomic, proteomic and immunoproteomic platforms in interpreting the response to live-attenuated vaccines.

Comparison of the vaccine antigen content and immunologic responses to those observed with wild-type infection could inform the design, optimization, and engineering of next-generation ETEC vaccines to enhance protective efficacy. Moreover, the platforms used in the present studies could be generalized to interrogation of live-attenuated vaccines for other important pathogens.

## Methods

### Bioinformatics and comparative genomics of ETEC isolates

To select candidate genes for protein expression, we analyzed the previously sequenced genomes of three parental ETEC isolates WS_1858B, WS_2773E, and WS3504D, used in the construction of ACE527,^13^ the genome of *E. coli* H10407^39^ the challenge strain used in these studies, and the genomes of a diverse group of clinical isolates. Data describing these strains is presented in supplementary table 1. The genome content of these isolates was compared using Large-Scale BLAST Score Ratio Analysis^40^ and encoded products having a signal for potential secretion to the surface were identified using PSORT,^41^ TMHMM,^42^ and SignalP.^43^ The resulting dataset includes 800 antigens identified in H10407, 157 antigens present in one or more of the three isolates of the ACE527 vaccine lacking in the H10407 genome, and an additional 4168 features identified in comparative analysis of 207 clinical ETEC isolates (supplementary data s1).^44,45^ The selected 4168 gene features were present in more than 40% of the ETEC isolates and were not present in the genomes of three common *E. coli* commensal isolates *E. coli* HS (GenBank Accession number NC_009800), *E. coli* K-12 (GenBank Accession number NC_007779.1), *E. coli* ATCC8739 (GenBank Accession number NC_010468) or *E. coli* IAI1 (GenBank Accession number NC_011741). Gene identifiers, DNA and predicted peptide sequences, and the isolates used as the template for isolation are included in supplementary_data_s1. Informatically selected features encompassed known ETEC antigens including the A and B subunits of heat-labile toxin (LT-A, and LT-B),CFs, the EatA^19^ serine protease, the EtpA adhesin,^37^ and the metalloprotease YghJ^46^ in addition to conserved and serotype specific regions of flagellin molecules represented in the challenge and vaccine strains.

Rapid Annotation using Subsystem Technology (RASTtk v 1.3.0, http://rast.nmpdr.org) ^47^was used to query completed ACE527 genomes for specific virulence factors. Multiplex PCR was used to verify the toxin profiles of parent and ACE527 vaccine strains using primers for *estH*, *estP*, and *eltB* encoding the STh, STp, and the B subunit of LT, respectively.^48^ PCR was also used to verify the presence or absence of the *eatA* and *etpA* genes.^49^ (supplementary table 2). SerotypeFinder v 2.0 (https://cge.cbs.dtu.dk/services/SerotypeFinder/) was used to assign H serotypes from whole genome sequence data.^50^

### Microarray construction

Genes encoding candidate ETEC surface-expressed antigens were amplified by PCR, cloned into pXI T7,^12,51^ and expressed in a cell-free in vitro transcription—translation (IVTT) system as previously described.^12^ Each IVTT protein included 5′ polyhistidine (HIS) and 3′ hemagglutinin (HA) epitopes. After robotic microarray printing onto nitrocellulose-coated glass slides, random slides were validated by probing with anti-His (mouse monoclonal clone HIS-1, Sigma-Aldrich, H1029-100UL) and anti-HA (rat monoclonal to HA peptide YPYDVPDYA, clone 3F10, Sigma Aldrich, 11867423001) followed by fluorescent secondary antibodies.

Recombinant antigens including the EtpA adhesin,^52^ the passenger domain of EatA,^19^ YghJ,^46^ antigen 43,^53^ EaeH,^54^ LT-A and LT-B, and flagellin (FliC)^29^ subunits were produced at Washington University in Saint Louis as previously described. Purified CFs, and CS antigens were produced at the Naval Medical Research Center, Silver Spring Maryland.

### Microarray antigen content

The microarrays used in this study were comprised of IVTT expressed proteins selected above and purified proteins representing known ETEC antigens (*n* = 38) (Supplementary dataset s1). Also included on the array are IVTT control spots (*n* = 28), positive control spots for IgG secondary antibody (*n* = 16), positive controls for human IgG (*n* = 16), positive control spots for IgA secondary antibody (*n* = 16), and positive controls for human IgA (*n* = 16).

### Vaccination with ACE527 and controlled human infection studies

Samples analyzed in the present study were derived from Phase 1/2b trial of ACE527 conducted in healthy human volunteers at the Centre for Immunization Research at Johns Hopkins University School of Public Health (clinical trial number NCT01739231).^27^ In this earlier trial, subjects were randomly assigned to three groups: the placebo group or groups that received three doses of ACE527 (~10^10^ cfu/dose) given orally with or without the dmLT adjuvant (25 μg per dose). At 6–7 months following the primary immunization, placebo and vaccine ± dmLT immunization subjects were rescreened for eligibility. Those enrolled were challenged with ~2 x 10^7^ colony forming units (cfu) of the H10407 ETEC challenge strain after an overnight fast, and assessed in an inpatient setting for development of ETEC-associated diarrhea and other signs and symptoms of enteric illness. The challenge phase of this study included 13 volunteers who received three oral doses of ACE527, 13 volunteers who received three oral doses ACE527 adjuvanted with 25 µg of dmLT, and 10 placebo recipients (supplementary dataset s1).

### ETEC strain validation

Strains used in these studies are summarized in supplementary table 1. The H10407 clinical challenge strain used in these studies was derived from cGMP Batch Production Record (BPR)-285-01, Lot 1514, manufactured on 15 October 2008 at the Walter Reed Army Institute of Research (WRAIR) Pilot Bio-production Facility, Silver Spring, Maryland. cGMP aliquots of vaccine strains ACAM2022 (BPR-966-00, lot 1621, manufactured 08 March, 2010), ACAM 2025 (BPR-977-00, lot 1633, manufactured 03 May, 2010), and ACAM2027 (BPR-969-00, lot 1625, manufactured 22 March, 2010) were obtained as lyophilized frozen stocks from WRAIR. Parent ETEC isolates WS-2773E, WS-3504D, and WS-1858B used to engineer the vaccine strains were forwarded from the University of Maryland. The toxin profile of each isolate was confirmed by multiplex PCR using primers for *eltB, estH*, and *estP;* and *etpA* and *eatA* genes fragments were amplified as described previously (supplementary table 2). To verify production of secreted proteins, immunoblotting for EtpA, EatA, and YghJ was performed as previously described.^55^ All immunoblots derive from the same experiment and were processed in parallel. Immunoblotting used affinity purified cross absorbed rabbit polyclonal antibodies against each of the respective recombinant proteins. The secondary antibody used for detection was goat anti-rabbit antibody conjugated to IRDye 680RD. (Catalogue number 925-68071).

### Production of subcellular fractions and proteome analysis

In characterizing the proteomes of the ACE527 vaccine strains and parents, subcellular fractions including outer membrane proteins (OMPs), outer membrane vesicles (OMV), and concentrated culture supernatants were prepared for tryptic digestion and peptide analysis using high resolution tandem mass spectrometry interfaced to *nano*-liquid chromatography (LC-MS/MS) as recently described for H10407.^12^ LC-MS data were used to search (MASCOT, version 2.5.1, Matrix Science, London, UK) conceptionally translated genomes of parent and vaccine strains (see bioproject PRJEB2286). The complete set of mass spectrometry data is included in supplementary dataset s2.

### Antibody lymphocyte supernatants

ALS were prepared from whole blood by isolation of PBMC, followed by in vitro culture of the PBMCs as previously described.^24^ The resulting supernatants were then stored at −80 °C for future use in these studies.

### Statistical analysis

Array comparisons were reported as the proportion of subjects with at least a 50% increase in normalized signal intensity between day 0 and day 7. *p* values reported in tables represent *t*-test comparisons of average normalized signals obtained for the two represented groups. In presentation of graphs where multiple comparisons are made, ANOVA was used to compare normalized signal data with a Kruskal–Wallis test for multiple comparisons.

### Human studies

Use of the archived biospecimens, and data in the present study was performed with the approval of the Institution Review Boards of Johns Hopkins University School of Medicine and Washington University School of Medicine.

### Reporting summary

Further information on research design is available in the Nature Research Reporting Summary linked to this article.

## Supplementary information

supplementary_information

reporting summary

supplementary_dataset_1

supplementary_dataset_2

supplementary_dataset_3

## Data Availability

Proteomics data have been uploaded to the ProteomeXchange^56,57^ database accessible at http://www.proteomexchange.org/ via accession number PXD014724. Protein Microarray data have been uploaded to the GEO database^58,59^ https://www.ncbi.nlm.nih.gov/geo/ under accession number GSE134792. Source data for the figures and tables is also provided in the supplementary information.

## Supplementary information

**Supplementary information** accompanies the paper on the *npj Vaccines* website (10.1038/s41541-019-0131-7).
