# Supplementary material for: Interrogation of a live-attenuated enterotoxigenic Escherichia coli vaccine highlights features unique to wild-type infection
Source: NPJ Vaccines. 2019 Aug 28;4:37. doi: 10.1038/s41541-019-0131-7 (PMC6713706; doi:10.1038/s41541-019-0131-7)

Supplementary figures

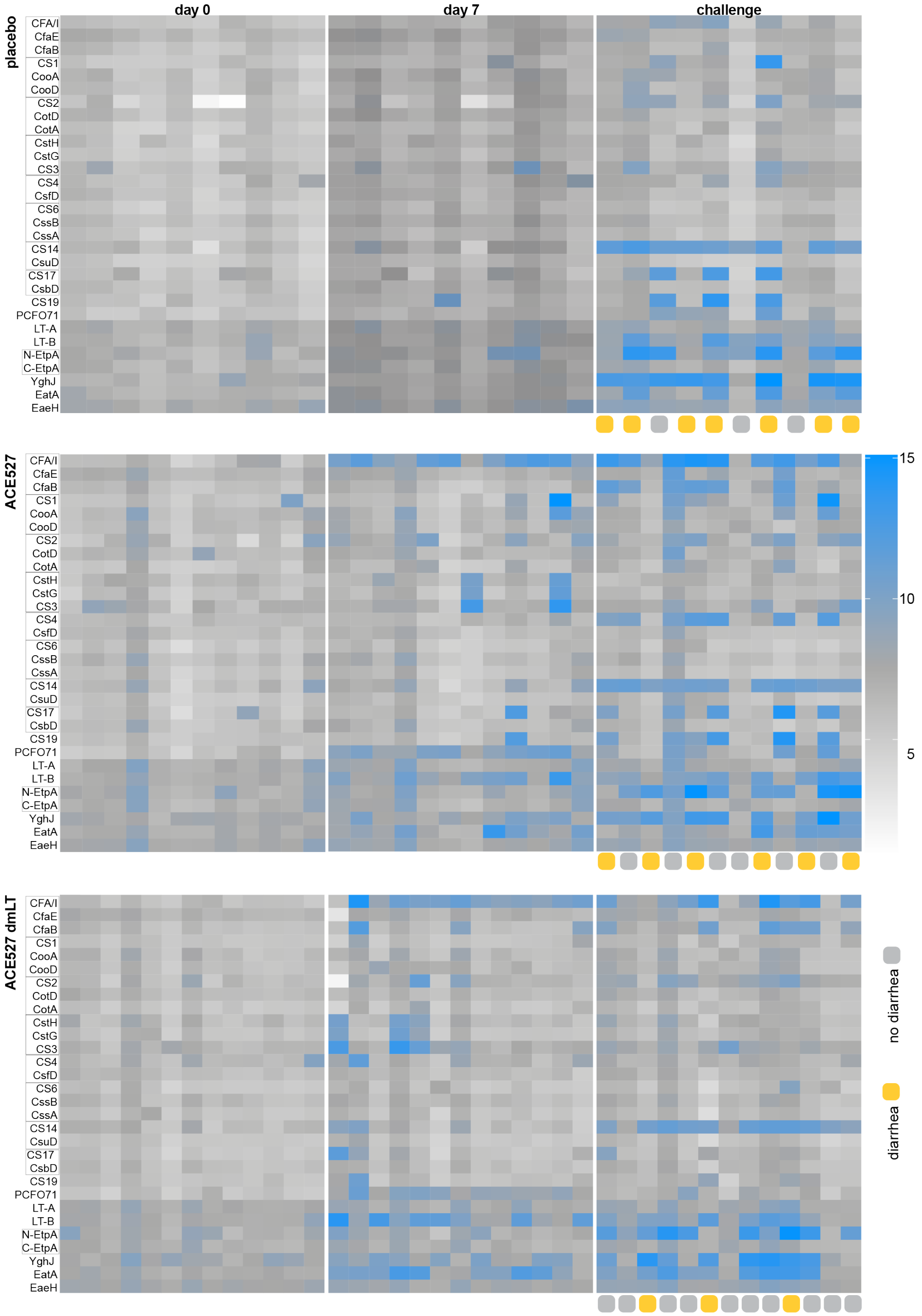

Supplementary figure 1

Antibody lymphocyte supernatant (ALS), IgA responses to recombinant antigens following vaccination with ACE527±dmLT, and challenge with H10407. Individual recombinant antigens are shown at left while individual volunteer data are arranged in columns. Data legend depicts log2 values of array signal intensity.

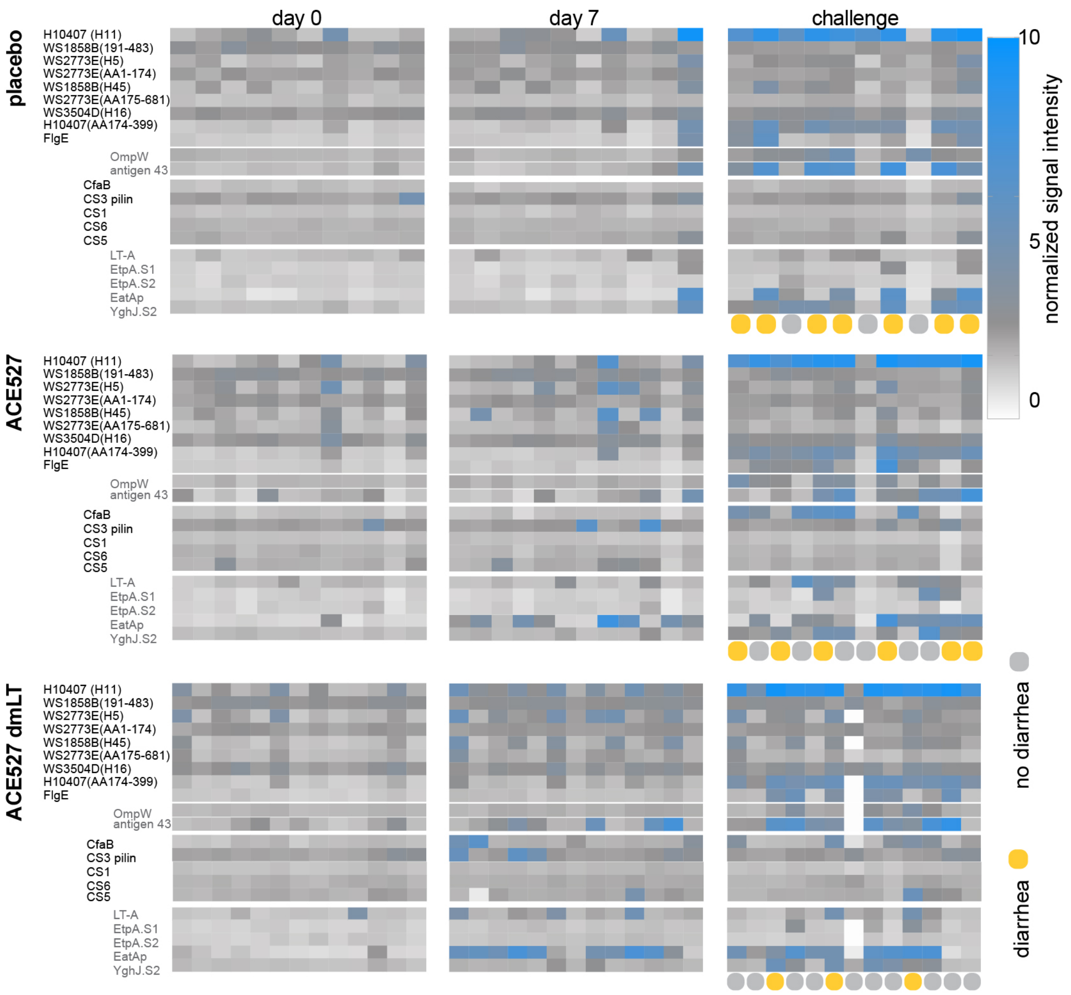

Supplementary figure 2.

Antibody lymphocyte supernatant (ALS) IgA responses to select IVTT antigens following vaccination with ACE527, ACE527 + dmLT, or placebo. Shown are responses prior to vaccination, day 7 post vaccination, and day 7 post challenge with ETEC H10407. Antigens are grouped by row according to function (flagellar antigens at top; followed by membrane proteins OmpW, and antigen 43; CF/CS colonization factor antigens; secreted proteins at bottom). Columns in each group represent individual volunteers (placebo n=10; ACE527 n=12; ACE527 +dmLT n=13). Volunteers experiencing diarrhea after challenge with ETEC H10407 are represented as yellow circles while those without diarrhea post challenge are shown as grey circles. Legend values at right indicate normalized signal intensity.

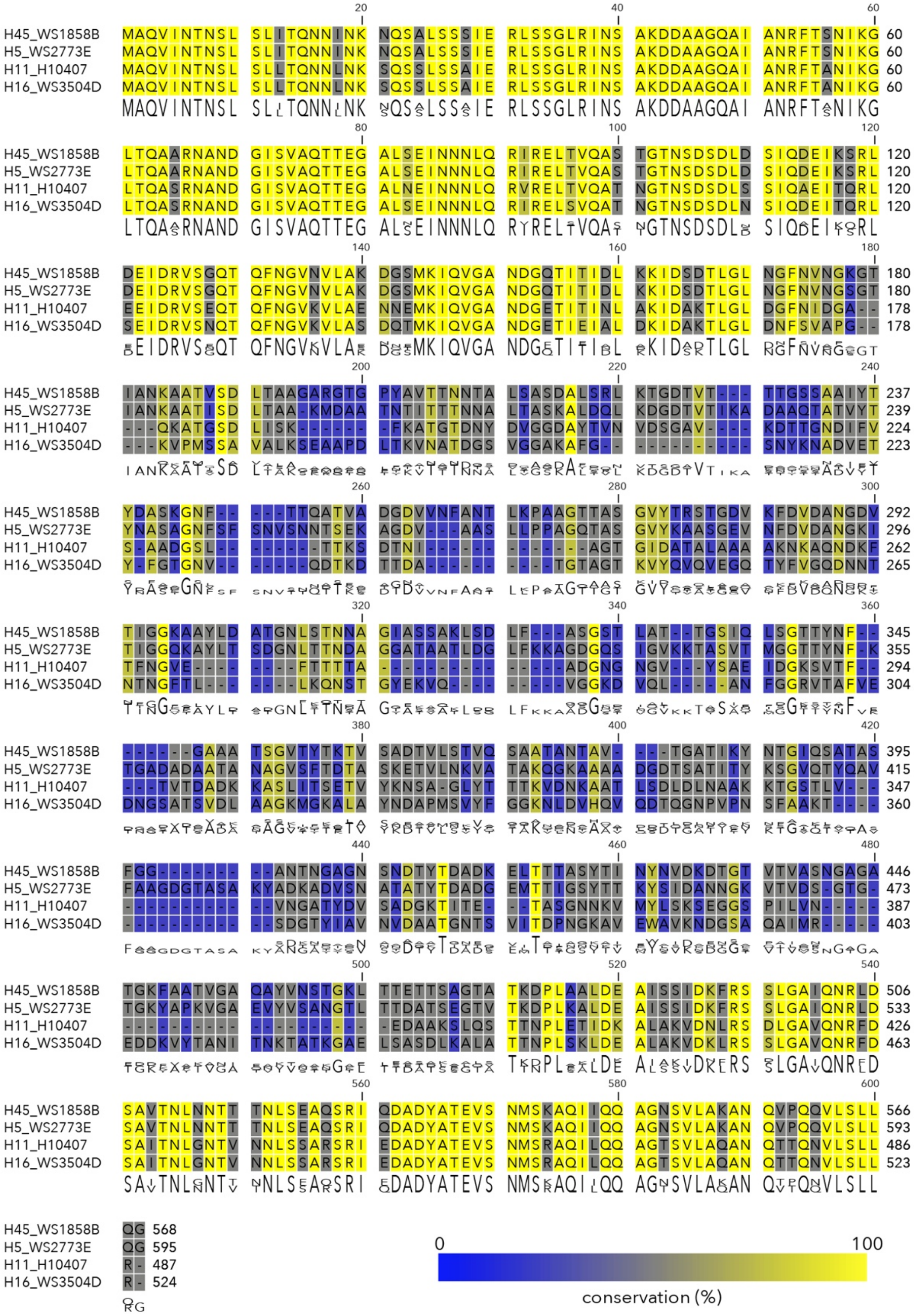

Supplementary figure 3.

MUSCLE[7] alignment of H10407 flagellin (H11 sequence) with flagellin (FliC) peptides from parental ACE527 strains WS1858B (H45), WS2773E (H5), and WS3504D (H16). MUSCLE v3.8.31 is available at http://www.drive5.com/muscle/.

Supplementary tables

Supplementary table 1

Supplementary table 1. H10407 challenge, ACE527 parental and vaccine strains

strain	genotype	description	reference
live attenuated vaccine parents and constructs			
WS-2773E	O141:H5 CS5 CS6 LT ST EAST1	(parental clinical isolate)	[1]
ACAM2022	O141:H5 CS5 CS6 Δ*astA* Δ*eltAB* Δ*estA* Δ*ompF::Ptac-LTB* Δ*aroC* Δ*phage*	vaccine isolate from WS-2773E	[1]
WS-3504D	O39:H16[a] CS2 CS3 LT ST EAST1 Ap^r	parental clinical isolate	[1]
ACAM2027	O39:H16 CS2 CS3 Δ*astA* Δ*eltAB* Δ*estA* Δ*ompC::CS1* Δ*ompF::Ptac-LTB* Δ*aroC*	vaccine isolate from WS-3504D	[1]
WS-1858B	O71:H45[b] CFA/I ST EAST1 Ap^r Tp^r Su^r	parental clinical isolate	[2]
ACAM2025	O71:H45 CFA/I Δ*astA* Δ*eltAB* Δ*estA* Δ*ompC* Δ*ompF::pLLTB*	vaccine isolate from WS-1858B	[1]
clinical challenge strain			
H10407	O78:H11 CFA/I LT/ST/EAST1	wild type-challenge strain	[3]

[a]Predicted by DNA sequence; Original serotype H12.
[b]Predicted by DNA sequence, original serotype H^-.
https://cge.cbs.dtu.dk/services/SerotypeFinder/[4]

supplementary table 2 primers used in these studies

primer designation	sequence 5′-3′	description	amplicon size (bp)	Reference:
jf082213.1	ATGTGCTTTG GCAGGTTAAT	*eatA* forward primer	1934	[5]
jf082213.2	ATATCCAGTC AGCACCCACT	*eatA* reverse primer	1934	[5]
jf082213.3	GGTTCAGGCA GTATCCAGAC	*etpA* forward primer	999	[5]
jf082213.4	GGTGTAGCTG TCTGACCACA	*etpA* reverse primer	999	[5]
jf092313.7	TACAAGCAGGATTACAACAC	ST-H reverse primer bp 205-186 of st1b gene	64	[6]
jf092313.8	AGTGGTCCTG AAAGCATG	ST-H forward primer bp 146-159 of st1b	64	[6]
jf092313.5	TCTTTCCCCTCTTTTAGTCAG	ST-P forward primer	166	[6]
jf092313.6	ACAGGCAGGA TTACAACAAA G	ST-P reverse primer	166	[6]
jf092313.3	ACGGCGTTAC TATCCTCTC	LT-forward primer binds 31-49 of LT-B gene	274	[6]
jf092313.4	TGGTCTCGGTCAGATATGTG	LT-reverse primer binds 304-285 of LT-B gene	274	[6]

Supplementary table 3 genetic compostion of ACE527 vaccine strains and parents

Supplementary table 3. genetic composition of ACE527 vaccine strains and parents[1]						
	WS2773E	ACAM2022	WS1858B	ACAM2025	WS3504D	ACAM2027
gene/locus						
colonization factors						
cfaA			+	+		
cfaB			+	+		
CS1						+
CS2					+	+
CS3					+	+
CS5	+	+				
CS6	+	+				
CS21			+	+	+	
toxins						
eltA	+				+	
eltB	+	+		+	+	+
estH	+				+	
astA	+				+	
estP						
secreted virulence antigens						
etpBAC			+		+	
eatA	+	+	+		+	+
yghJ	+	+	+	+	+	+
cexE			+	+		
clyA	+	+	+	+	+	+
cell surface antigens						
tia						
tibA						
fimH	+	+	+	+		
fimA	+	+	+	+		
ecpA	+	+				
antigen 43	+	+	+	+	+	+
motility						
fliC	+	+	+	+	+	+
flgE	+	+	+	+	+	+
toxin secretion						
T2SS[2] (Gsp)	+	+	+	+	+	+
tolC	+	+	+	+	+	+

[1]from whole genome sequence analysis of ACE527 parents and vaccine strains using RASTtk [28]

[2]T2SS = type 2 secretion system General Secretion Pathway genes *pppA* through *gspM*

Supplementary references

1. Turner AK, Stephens JC, Beavis JC, et al. Generation and characterization of a live attenuated enterotoxigenic Escherichia coli combination vaccine expressing six colonization factors and heat-labile toxin subunit B. Clin Vaccine Immunol **2011**; 18:2128-35.
2. Turner AK, Beavis JC, Stephens JC, et al. Construction and phase I clinical evaluation of the safety and immunogenicity of a candidate enterotoxigenic Escherichia coli vaccine strain expressing colonization factor antigen CFA/I. Infect Immun **2006**; 74:1062-71.
3. Evans DJ, Jr., Evans DG. Three characteristics associated with enterotoxigenic Escherichia coli isolated from man. Infect Immun **1973**; 8:322-8.
4. Joensen KG, Tetzschner AM, Iguchi A, Aarestrup FM, Scheutz F. Rapid and Easy In Silico Serotyping of Escherichia coli Isolates by Use of Whole-Genome Sequencing Data. J Clin Microbiol **2015**; 53:2410-26.
5. Del Canto F, Valenzuela P, Cantero L, et al. Distribution of Classical and Nonclassical Virulence Genes in Enterotoxigenic Escherichia coli Isolates from Chilean Children and tRNA Gene Screening for Putative Insertion Sites for Genomic Islands. J Clin Microbiol **2011**; 49:3198-203.
6. Rodas C, Iniguez V, Qadri F, Wiklund G, Svennerholm AM, Sjoling A. Development of multiplex PCR assays for detection of enterotoxigenic Escherichia coli colonization factors and toxins. J Clin Microbiol **2009**; 47:1218-20.
7. Edgar RC. MUSCLE: a multiple sequence alignment method with reduced time and space complexity. BMC Bioinformatics **2004**; 5:113.

PCR Results for EatA and EtpA

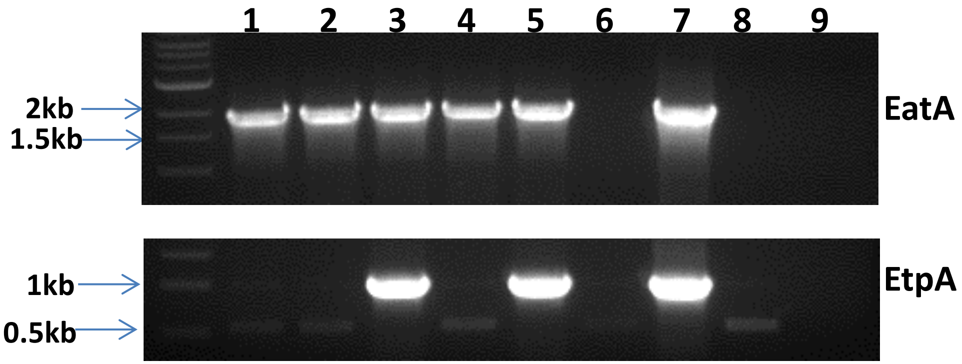

Well #	jf #	Sample ID	Well #	jf #	Sample ID
1	2864	WS2773E	6	2904	ACAM2025
2	2900	ACAM2022	7	H10407	Positive control
3	2866	WS3504D	8	MG-1655	Negative control
4	2902	ACAM2027	9	Water	Negative control
5	2862	WS1858B			

DNA template: genomic DNA
Primers: EatA (082213. 1&2) and EtpA (082213. 3&4)
Products' size: EatA=1934bp EtpA=999bp

8/27-28/18

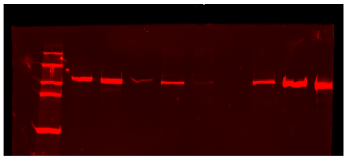

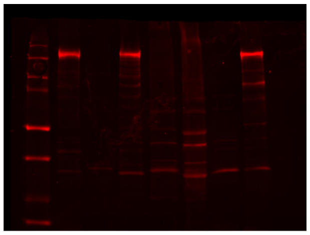

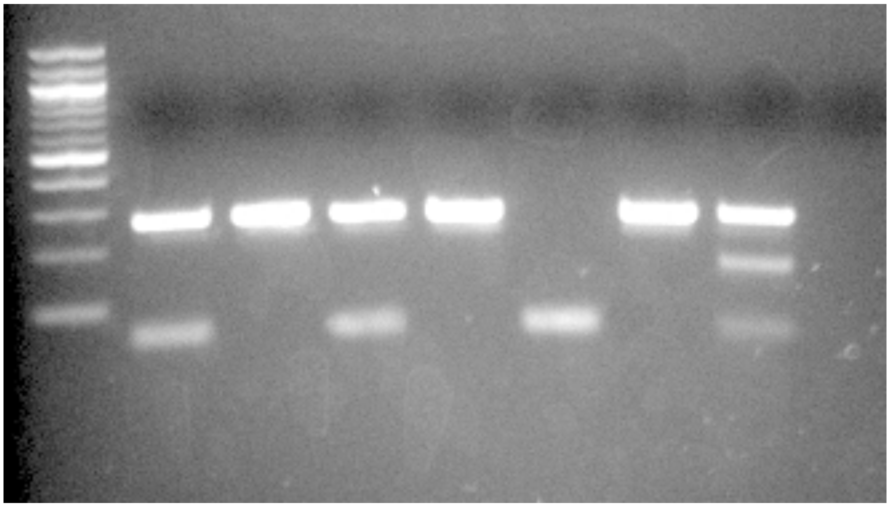

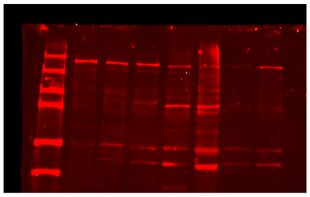

Supplement: Supplementary file 1 — supplementary_information [file 41541_2019_131_MOESM1_ESM.pdf]
